# MiR-612, miR-637, and miR-874 can Regulate VEGFA Expression in Hepatocellular Carcinoma Cell Lines

**DOI:** 10.3390/genes13020282

**Published:** 2022-01-30

**Authors:** Márcia Maria U. Castanhole-Nunes, Nathalia M. Tunissiolli, André R. C. P. Oliveira, Marlon F. Mattos, Ana Lívia S. Galbiatti-Dias, Rosa S. Kawasaki-Oyama, Erika C. Pavarino, Renato F. da Silva, Eny M. Goloni-Bertollo

**Affiliations:** 1 Research Unit of Genetics and Molecular Biology (UPGEM), Department of Molecular Biology, Faculty of Medicine of Sao Jose do Rio Preto (FAMERP), Sao Jose do Rio Preto 15090-000, SP, Brazil; mcastanhole@gmail.com (M.M.U.C.-N.); nathaliatunisiolli@yahoo.com (N.M.T.); rodrigueiro@gmail.com (A.R.C.P.O.); marlon.fraga.mattos@outlook.com (M.F.M.); analiviagalbiattidias@gmail.com (A.L.S.G.-D.); rosa.oyama@famerp.br (R.S.K.-O.); erika@famerp.br (E.C.P.); 2 Department of Surgery and Liver Transplantation, Hospital de Base/FUNFARME/FAMERP-Foundation Faculty of Medicine of Sao Jose do Rio Preto, Sao Jose do Rio Preto 15090-000, SP, Brazil; renatosilva@famerp.br

**Keywords:** microRNAs, gene expression, liver neoplasms, angiogenesis, cancer biomarkers, transfection

## Abstract

MicroRNAs (miRNAs) are short non-coding RNA molecules acting as important posttranscriptional gene and protein expression regulators in cancer. The study goal was to examine VEGFA (vascular endothelial growth factor A) expression in hepatocellular carcinoma (HCC) cell lines upon transfection miR-612, miR-637, or miR-874. **Methods:** MiR-612 mimics, miR-637 mimics, or miR-874 inhibitors were transfected using Lipofectamine RNAiMax in both HCC cell lines, HepG2 and HuH-7. Real-time PCR, Western blotting, and ELISA methods were used to evaluate VEGFA regulation by the miRNAs. **Results:** Gene and protein expression levels of VEGFA were down-expressed in both cell lines, HepG2 and HuH-7, transfected with miR-612 or miR-637. Transfection with miR-874 inhibitor showed an increase in VEGFA gene expression in HepG2 and HuH-7 cell lines; however, no regulation was observed on VEGFA protein expression by miR-874 inhibition. Correlation analysis between miRNAs and VEGFA protein expression showed that miR-637 and miR-874 expression present inversely correlated to VEGFA protein expression. **Conclusions**: VEGFA was down-regulated in response to hsa-miR-612 or hsa-miR-637 overexpression; however, the modulation of VEGFA by miR-874 was observed only at the gene expression and thus, needs further investigation.

## 1. Introduction

Hepatocellular carcinoma (HCC) is the most common primary liver malignancy and is a leading cause of cancer-related deaths worldwide [1]. The main risk factors include chronic infection with the hepatitis B or hepatitis C virus, consumption of aflatoxin-contaminated foods, heavy alcohol intake, obesity, smoking, and diabetes [2]. HCC is more prevalent in males than in females (ratio 1:2.4), with high occurrence rates in eastern and southern Asia. As estimated in 2012 by GLOBOCAN, the ratio of incidence and mortality of HCC is 0.95, indicating a poor prognosis [3].

MicroRNAs (miRNAs) are short non-coding RNA molecules acting as important posttranscriptional gene and protein expression regulators [4,5,6,7]. Some miRNAs can regulate the vascular endothelial growth factor A (VEGFA) gene, which acts in blood vessel growth in some cancer types, including hepatocellular carcinoma [8,9,10,11,12]. Studies in cancer, including HCC, have shown that miRNAs have an essential role in angiogenesis, tumorigenesis, and metastasis [13,14,15]. A review highlighted that miRNAs are meaningful for regulating particular endothelial processes downstream of VEGF, thus representing therapeutic targets involved in the VEGF ligand-receptor interaction or VEGFR kinase activity [9].

VEGF is a growth factor that activates receptor tyrosine kinases, initiating the RAS-RAF-MEK (Map kinase)-ERK (extracellular signals regulated kinase) MAPK (Mitogen-activated protein kinase) signaling cascade. This cascade induces key transcription factors as well as the epithelial-mesenchymal transition that results in cell motility and invasion [16]. VEGFA products in cancer have been studied with their interaction in different signaling pathways, such as STAT3 (activator of transcription 3), KRAS (Kirsten rat sarcoma virus), and MAPK, mediated by protein kinase B (PI3K) and regulated by ERK, among other factors. These interactions were observed in different types of cancer, such as liver and lung. In some cases, the activation of these pathways is related to tumor aggressiveness and, therefore, regulation through miRNAs [16,17]. Evidence has suggested that the regulation of miRNAs in cancer can identify biomarkers for the diagnosis and treatment of cancer [7,12]. Because the inhibition of VEGFA signaling interferes in the angiogenesis process, and miRNAs may provide a potential anti-angiogenesis therapy for cancer treatment, we evaluated the expression of VEGFA in HCC cell lines upon treatment with miR-612 and miR-637 mimics and miR-874 inhibitors.

## 2. Materials and Methods

### 2.1. Cell Lines

Hepatoma cell line HepG2, derived from a liver HCC of a 15-year-old Caucasian male [18], and HuH-7 cell line from HCC taken from a liver tumor of a 57-year-old Japanese male [19] were used in the study. Both cell lines were cultured in DMEM (Dulbecco’s modified Eagle’s medium) (Cultilab, Campinas, São Paulo, BR) supplemented with 10% fetal bovine serum (Cultilab, Campinas, São Paulo, BR), 100 U/mL sodium penicillin, 100 mg/mL streptomycin (Cultilab, Campinas, São Paulo, BR), and 1% L-glutamine (Cultilab, Campinas, São Paulo, BR) at 37 °C in a 5% CO_2_ atmosphere.

### 2.2. MiRNA Prediction

Seventeen miRNAs predicted by the miRNAs databases, miRBASE (http://www.mirbase.org/ (accessed on 20 March 2017)), TargetScan (http://www.targetscan.org/vert_71 (accessed on 20 March 2017)), and DIANA-TarBase v7.0 databases (http://diana.imis.athena-innovation.gr/DianaTools (accessed on 16 July 2017)) in our previous study were selected [20]. A previous study analyzing relative miRNA expression in 40 samples (HCC and non-tumor tissue) showed that 9 out of 17 predicted miRNAs were differentially expressed in tumor tissues compared to the control. The relative VEGFA gene expression showed a relationship to the miRNA expression [20]. In the present study, we used the mimics of low expressed miR-612, miR-637, and an inhibitor of highly expressed miR-874 to study the regulation of the VEGFA target gene.

### 2.3. Transfection of miRNAs in HepG2 and HuH-7 Cell Lines

Transfection assays of the mirVana™ inhibitor for hsa-miR-874 (MH12355, Thermo Scientific, Waltham, Massachusetts, EUA), mirVana™ hsa-miR-612 mimics (MC11461, Thermo Scientific, Waltham, Massachusetts, EUA), and mirVana™ hsa-miR-637 mimics (MC11545, Thermo Scientific, Waltham, Massachusetts, EUA) were conducted using Lipofectamine RNAiMax (Invitrogen, Waltham, Massachusetts, EUA), following the manufacturer’s instructions. To determine the best concentration values of the mirVana™ inhibitor, mirVana™ mimics and Lipofectamine RNAiMax were performed concentration tests. Subsequently, the cells were cultured for 48 h in 100 μL of Opti-MEM serum-free medium (Invitrogen, Waltham, Massachusetts, EUA), 1 μL of Lipofectamine RNAiMax (Invitrogen, Waltham, Massachusetts, EUA), and 10 mM of miR-874 inhibitor, miR-612, or miR-637 mimics. Three independent experiments were performed. After this, the RNA was extracted to verify the efficiency of transfection using the respective positive and negative control genes and miRNAs by qPCR (Quantitative real-time PCR). The positive controls for inhibitor assay were the HMGA2 gene and let-7c miRNA. Similarly, the TWF1 gene and miR-1 were positive controls for mimic assays.

### 2.4. RNA and miRNA Extraction

After transfection, RNA and miRNA were extracted from HepG2 and HuH-7 cell lines using Trizol reagent (Invitrogen, Waltham, Massachusetts, EUA). Complementary DNA (cDNA) was synthesized using the High Capacity cDNA reverse transcription Kit (Thermo Fisher Scientific, Waltham, Massachusetts, EUA). The cDNAs of miRNAs were synthesized using Taqman MicroRNA Reverse Transcription Kit (Applied Biosystems, Waltham, Massachusetts, EUA).

### 2.5. Quantitative Real-Time PCR for Expression of VEGFA Gene and miRNAs

Expression analysis of the VEGFA (Hs00900055_m1) gene and miR-612 (001579), miR-637 (003307), and miR-874 (00268) was performed by qPCR using specific TaqMan probes (Thermo Fisher Scientific, Waltham, Massachusetts, EUA) on the CFX 96 Real-Time System (Bio-Rad, Hercules, California, EUA). All reactions were performed in duplicate and included a contamination control. The genes GAPDH (Hs03929097_g1) and RPLPO (4333761F) were used as reference genes for the normalization of VEGFA expression data. The genes U6 (001973) and RNU48 (001006) were used for normalization of miR-612, miR-637, and miR-874 expression data (Thermo Fisher Scientific, Waltham, Massachusetts, EUA). Relative quantification (RQ) of genes and miRNA expression levels in HCC lineages were calculated using the 2^-ΔΔCt^ method concerning the negative control [21].

### 2.6. Extraction and Quantification of Protein

The proteins were extracted using the RIPA Buffer (Sigma-Aldrich, San Luis, Missouri, EUA), and quantified using the Pierce ^TM^ BCA Protein Assay kit (Thermo Fisher Scientific, Waltham, Massachusetts, EUA). Quantification of VEGFA protein in the transfection assays with miR-612, miR-637, miR-874, and negative control was performed using the VEGFA Duo Set ELISA Kit (R&D Systems, Minneapolis, Minnesota, EUA) and Western blotting (WB) method utilizing Anti-VEGFA antibody (ab1316, Abcam, Cambridge, United Kingdom), following the manufacturer’s instruction.

### 2.7. Statistical Analysis

The statistical analysis was performed using GraphPad Prism software version 6. Continuous data distribution was evaluated using D’Agostino and Pearson’s normality test. Student’s *t* test, Wilcoxon Signed rank test, and Mann–Whitney test were used to evaluate the VEGFA gene and protein expression data. The correlation between the expression of miRNAs and VEGFA proteins was analyzed by Spearman’s correlation or Pearson’s correlation. Values of *p* < 0.05 were considered significant.

## 3. Results

### 3.1. Bioinformatics-TCGA Analysis

**Figure 1:**
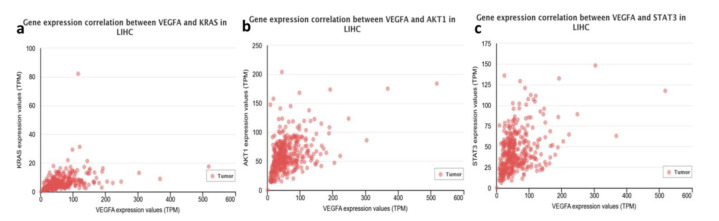
Pearson positive correlation of VEGFA expression with (**a**) KRAS (R = 0.36), (**b**) AKT1 (R = 0.45), and (**c**) STAT3 (R = 0.4) in TCGA analysis in LIHC [22].

The analysis of The Cancer Genome Atlas Program (TCGA) database through the UALCAN [22] website showed that there is a positive correlation between KRAS (Figure 1a), AKT1 (Figure 1b), and STAT3 (Figure 1c) genes in Liver Hepatocellular carcinoma (LIHC) and VEGFA expression.

**Figure 2:**
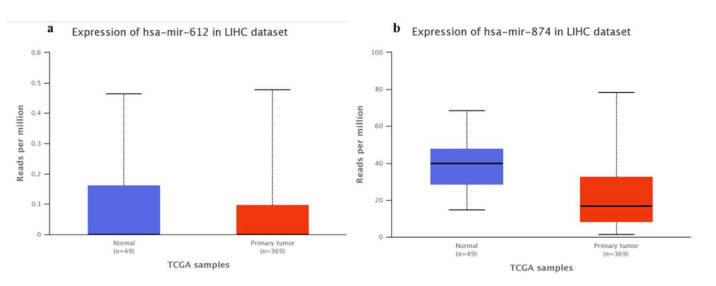
TCGA expression data of (**a**) hsa-miR-612 (*p* = 0.52) and (**b**) miR-874-3p (*p* = 0.20) in LIHC, comparing normal tissue and primary tumor [22].

In addition, miR-612 (Figure 2a) and miR-874-3p (Figure 2b) expression analyzes were performed in Hepatocellular carcinoma (HCC), comparing normal tissues with primary tumors, allowing visualization of the expression profile in primary tumor tissue samples.

Regarding miR-637, it was not possible to obtain this information as there are no data in the TCGA for this microRNA in HCC.

### 3.2. Transfection Efficiency Test

The transfection efficiency was calculated by the relative expression levels of TWF1 in cells treated with mirVana ™ miRNA Mimic miR-1 positive control (Applied Biosystems, Waltham, Massachusetts, EUA) in comparison to the negative control, to be approximately 75% in the HuH-7 cell line and 65% in the HepG2 cell line. Transfection efficiency test for the inhibition assay using positive controls, mirVana™ miRNA Inhibitor let-7c (Applied Biosystems, Waltham, Massachusetts, EUA) for HMGA2 gene expression showed a 31% decrease in HepG2 cells and a 57% increase in HuH-7 cells. This divergence between the cell lines may be related to differences in the gene regulation by miRNA and warrants further evaluation.

### 3.3. MiR-874, miR-612, and miR-637 Expression in Transfected HCC Cells

The expression levels of all three miRNAs were found to be up-regulated as compared to the negative controls in both HCC cell lines. Following are the statistical values obtained for each miRNA in three independent experiments: miR-874 (HuH-7: *p* = 0.1250; median = 10.52; HepG2: *p* = 0.1250; median = 5663), miR-612 (HuH-7: *p* = 0.0313; median = 2.053; HepG2: *p* = 0.0313; median = 14.03), and miR-637 (HuH-7: *p* = 0.313; median = 6.115; HepG2: *p* = 0.313; mean = 12.34).

### 3.4. VEGFA Expression in Transfected HCC Cell Lines

We performed an assay to evaluate the influence of miR-612 and miR-637 on VEGFA gene expression and observed significant difference in the expression of this gene upon transfection with miR-612 and miR-637 mimics. Expression of VEGFA was lower in miR-637-transfected cells than in miR-612-transfected cells. In the HepG2 cell line, the VEGFA gene expression showed a 51% reduction upon transfection with miR-612 (RQ mean = 0.49, *p* < 0.0001), and a 48% reduction upon transfection with miR-637 (RQ mean = 0.52, *p* = 0.0004). In the HuH-7 cell line, the expression of VEGFA decreased by 4% in cells treated with miR-612 (*p* < 0.0001), and by 73% with miR-637 (*p* < 0.0001). All values were obtained by comparing them with the negative control.

**Figure 3:**
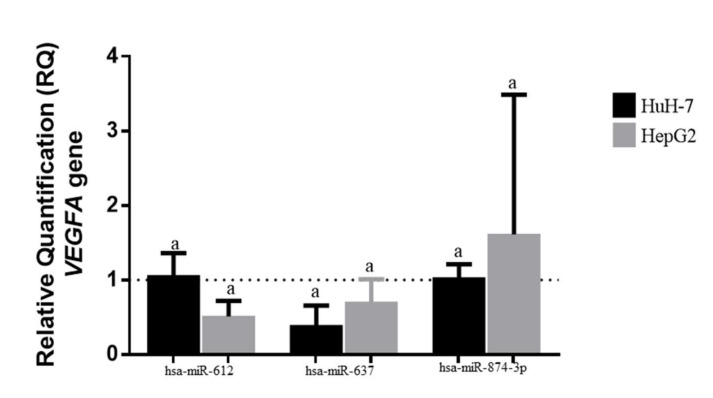
Relative quantification of VEGFA gene, transfected to miR-612, miR-637, and miR-874 on HepG2 and HuH-7 concerning the respective negative control (RQ = 1.00). a—Corresponding to significant *p* values.

Cells transfected with miR-874 inhibitor showed a 93% increase in VEGFA expression (RQ = 1.93, *p* = 0.0005) in the HepG2 cell line compared to the negative control, and equal or low expression in the HuH-7 cell line when compared to the control (RQ = 1.00, *p* < 0.0001) (Figure 3).

### 3.5. Protein Expression of VEGFA in Transfected HCC Cell Lines

**Figure 4:**
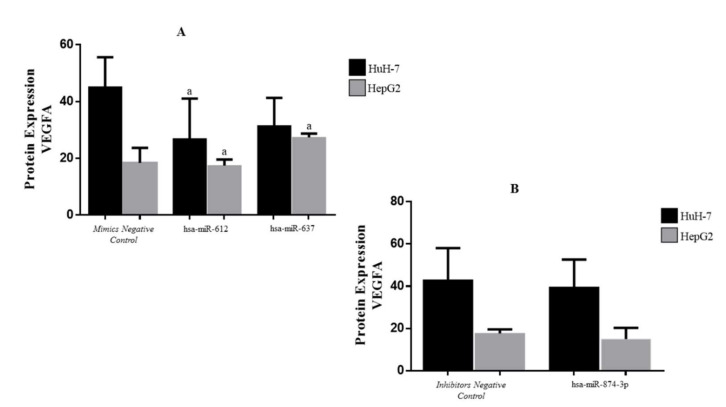
VEGFA protein expression compared to the respective negative control, transfected to miR-612, miR-637 (**A**), and miR-874 (**B**) on HepG2 and HuH-7 cell lines (ELISA method). a—Corresponding to significant *p* values.

**Figure 5:**
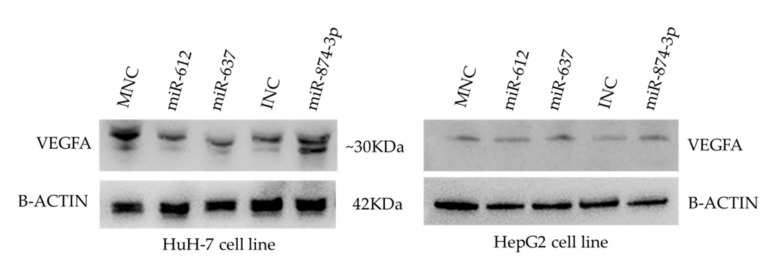
Western blotting analysis of VEGFA treated with the mimics negative control (MNC), hsa-miR-612, hsa-miR-637, inhibitor negative control (INC), and hsa-miR-874-3p, in the HuH-7 and HepG2 cell lines.

VEGFA protein quantification was performed by ELISA (Figure 4) and WB (Figure 5). WB results showed a reduced expression in miR-612 (HuH-7 cell line) and miR-637 (HuH-7 and HepG2 cell line). However, it was not possible to evaluate the transfection using miR-874-3p. ELISA methods showed the quantification after transfection with the miR-612 (HuH-7: *p* = 0.0106, mean= 26.90; HepG2: *p* > 0.9999, mean = 16.81) and miR-637 (HuH-7: *p* = 0.0168, mean = 31.54; HepG2: *p* = 0.0051, median = 23.30) mimics, and the miR-874 inhibitor (HuH-7: *p* = 0.6367, mean = 39.66; HepG2: *p* = 0.6222, median = 15.30). The protein expression was analyzed by comparing to the same target protein evaluated in transfection performed in the mimic negative controls (HuH-7: mean = 45.24; HepG2: median = 20.68) and inhibitor negative controls (HuH-7: mean = 43.04; HepG2: median = 17.73) (Figure 4).

The correlation between the gene and protein expression was not significant. However, the miRNA and protein expression correlation analysis showed that expression levels of miR-637 (R = -0.883 *p* = 0.0110) and miR-874 (R = -0.084 *p* = 0.0010) have an inverse correlation with VEGFA protein expression.

## 4. Discussion

In the present study, we utilized mimics for under-expressed miRNAs and inhibitors for overexpressed miRNA. In our preliminary study in HCC and non-tumor tissue samples (*n* = 40), we observed higher expression of VEGFA in tumor tissues than in non-tumor samples. Additionally, nine miRNA targets of VEGFA with relative differential expression were identified [20].

Our functional analysis showed that the VEGFA gene was down-regulated by hsa-miR-612 and hsa-miR-637; however, such effect was not observed upon inhibition of hsa-miR-874. VEGF is responsible for new blood vessel growth, and miRNA-mediated decreased expression of VEGF can contribute to angiogenesis inhibition [9].

VEGF is a growth factor that activates receptor tyrosine kinases, initiating the RAS-RAF-MEK (Map kinase)–ERK MAPK signaling cascade. This cascade induces key transcription factors as well as the epithelial-mesenchymal transition that results in cell motility and invasion [16]. The activated RAS-RAF-MEK pathway may be associated with metastasis and aggressive tumors in HCC. Interestingly, miR-612 levels are inversely correlated with HCC tumor size and stage, microvascular invasion, and intrahepatic metastasis, as well as the protein levels of AKT2 (AKT Serine/Threonine Kinase 2) and EMT biomarkers [23]. Tao et al. (2013) [23] showed that transfection of miR-612 in HCC cell lines resulted in increased E-cadherin, decreased vimentin, and EMT suppression, supporting the involvement of this miRNA in EMT through different regulators. Additionally, the authors suggested that reduced expression levels of miR-612 may promote EMT and metastasis in HCC [23].

In the present study, the VEGFA gene was shown to be down-regulated in both cell lines after transfection by miR-637. Zhang et al. (2011) [24] found that miR-637 might be a useful tool for therapy against HCC as it inhibits the activation of signal transducers, such as STAT3 [25]. Under hypoxic conditions, STAT3 is activated and promotes VEGF expression and angiogenesis [25], contributing to cell proliferation and tumorigenesis [26]. Thus, miRNA-mediated regulation of factors involved in hypoxia may contribute to the reduction of tumor progression [25].

MiR-874 Inhibition did not affect on the Huh-7 cell line in our study, once the expression gene in cell lines transfected was equal to the negative control. Probably, the concentrations of inhibitor used for transfection were not sufficient to observe the effect on VEGF gene regulation in this cell line. On the other hand, VEGF gene expression increased significantly in response to miR-874 inhibition in the HepG2 cell line. Zhang et al. (2015) [27] showed a negative correlation between miR-874 and the STAT3 gene in the gastric tumor, suggesting that the down-regulation of miR-874 can contribute to tumor angiogenesis through the STAT3/VEGFA pathway. The analysis of TCGA [22] data showed the VEGFA interaction positive correlation to KRAS, AKT1, and STAT3 genes (Figure 1). Furthermore, it was possible to observe the miR-612 and miR-874 down-expression in tumor samples compared to normal tissue [22], justifying that treatments with mimetic miRNAs would help to reduce the expression of genes involved in tumor development (Figure 2). No data on miR-637 expression in HCC tumor samples were observed, which is new data for the cancer database.

However, our protein expression data showed a reduction in VEGFA levels in both cell lines and a significant negative correlation between miR-874 and VEGFA protein in the HuH-7 cell line. This result may be attributed to the overexpression of miR-874, even after its inhibition. This possibly represents the characteristic of miRNAs posttranscriptional processing, where the transcribed gene is degraded by miR-874 overexpression before its translation [28]. Moreover, other factors may influence the VEGFA protein expression [29].

Because overexpression of miR-612 and miR-637 resulted in down-regulation of the VEGFA gene and protein in HepG2 and HuH-7 cell lines, we can conclude that these miRNAs can regulate VEGFA, while further studies are needed to better understand the miR-874 involvement in HCC. The present study provides information regarding miRNA-mediated regulation of VEGFA and suggests possible molecular mechanisms of liver carcinogenesis.

## 5. Conclusions

In conclusion, VEGFA is down-regulated in response to hsa-miR-612 and hsa-miR-637 overexpression; however, the modulation of VEGFA by miR-874 observed only at the gene expression level requires further investigation. The new regulatory data with miR-637 in liver cancer have an importance for characterization in the databases, being a potential biomarker for VEGFA.

## Data Availability

The data can be found at the Research Unit in Genetics and Molecular Biology (UPGEM), at the Faculty of Medicine of São José do Rio Preto (FAMERP).
